# An enhanced recovery after surgery pathway: LOS reduction, rapid discharge and minimal complications after anterior cervical spine surgery

**DOI:** 10.1186/s12891-022-05185-0

**Published:** 2022-03-15

**Authors:** Xue Leng, Yaqing Zhang, Guanzhong Wang, Libangxi Liu, Jiawei Fu, Minghui Yang, Yu Chen, Jiawei Yuan, Changqing Li, Yue Zhou, Chencheng Feng, Bo Huang

**Affiliations:** Department of Orthopedics Xinqiao Hospital, Army Medical University, 183 Xinqiao Main Street Shapingba, Chongqing, 400037 People’s Republic of China

**Keywords:** Enhanced recovery after surgery, Anterior cervical discectomy and fusion, Length of stay, Cost

## Abstract

**Background:**

Enhance recovery after surgery (ERAS) is a new and promising paradigm for spine surgery. The purpose of this study is to investigate the effectiveness and safety of a multimodal and evidence-based ERAS pathway to the patients undergoing anterior cervical discectomy and fusion (ACDF).

**Methods:**

The patients treated with the ACDF-ERAS pathway were compared with a historical cohort of patients who underwent ACDF before ERAS pathway implementation. Primary outcome was length of stay (LOS). Secondary outcomes included cost, MacNab grading, complication rates and 90-day readmission and reoperation. And perioperative factors and postoperative complications were reviewed.

**Results:**

The ERAS protocol was composed of 21 components. More patients undergoing multi-level surgery (*n* ≥ 3) were included in the ERAS group. The ERAS group showed a shorter LOS and a lower cost than the conventional group. The postoperative satisfaction of patients in ERAS group was better than that in conventional group. In addition, the rate of overall complications was significantly higher in the conventional group than that in the ERAS group. There were no significant differences in operative time, postoperative drainage, or 90-day readmission and reoperation.

**Conclusions:**

The ACDF-tailored ERAS pathway can reduce LOS, cost and postoperative complications, and improve patient satisfaction without increasing 90-day readmission and reoperation.

## Background

Enhanced recovery after surgery (ERAS), known as fast-track or rapid recovery surgery, is an integrated, multimodal and evidence-based approach to improve patient care and outcomes and was first introduced by Henrik Kehlet in 1997 [1]. The aim of ERAS is to minimize surgical stress responses, reduce the length of stay (LOS), decrease complications and improve patient experience [2, 3]. ERAS pathways have been widely implemented in numerous surgical areas [4–7]. For spine surgery, ERAS is a new and promising paradigm [6, 8].

Recently, two meta-analyses have shown that ERAS in spine surgery helps to reduce complications, readmissions, length of stay, cost and opioid use and improve patient-reported outcomes and functional recovery [9, 10]. The efficacy and safety of ERAS for patients undergoing elective spinal surgery have been recognized by practitioners. Given the protracted recovery phase of and intensive postoperative pain associated with invasive spine surgeries, incorporating ERAS into spine surgeries is an essential and natural next step. So far, the application of ERAS to degenerative lumbar spinal surgery, oncological spinal surgery and spinal deformity surgery has been reported [11–17].

With the aging of the population, the prevalence of degenerative cervical spine disease is increasing. The demand for cervical spine surgery is growing. Anterior cervical discectomy and fusion (ACDF) is widely used for the treatment of degenerative disease of the cervical spine resulting in central and neuroforaminal stenosis [18–23]. ACDF is a highly standardized procedure that has shown high efficiency and low mortality. However, the complication rates, LOS, cost and patient satisfaction of ACDF vary widely [24, 25]. Therefore, we are interested in establishing and implementing an ERAS pathway for ACDF to shorten LOS and improve outcomes.

Herein, we present an ERAS pathway for ACDF that we established and implemented. A retrospective study of patients undergoing ACDF was conducted. In this study, we introduce the ERAS pathway in detail and compare the outcomes of patients treated with ERAS care to the outcomes of patients treated with conventional care.

## Methods

### Study design

All experimental protocols were approved by the Ethics Committee of the Army Medical University and the IRB approval number is 2021-R.No. 069–01. Also, all methods were carried out in accordance with relevant guidelines and regulations and informed consent was obtained from all subjects. In our institution, ERAS was established and implemented progressively starting in January 2019. This is a retrospective analysis of collected data from all patients who underwent ACDF before (the conventional group, from December 2015 to December 2018) and after (the ERAS group, from January 2019 to July 2021) ERAS pathway implementation. Inclusion criteria included the patients with cervical spondylosis, spondylotic myelopathy and radiculopathy that were refractory to conservative treatment. Patients with prior cervical surgery, corpectomy, neoplasm, infection, trauma and deformity were excluded.

### ERAS pathway development and implementation

**Fig. 1:**
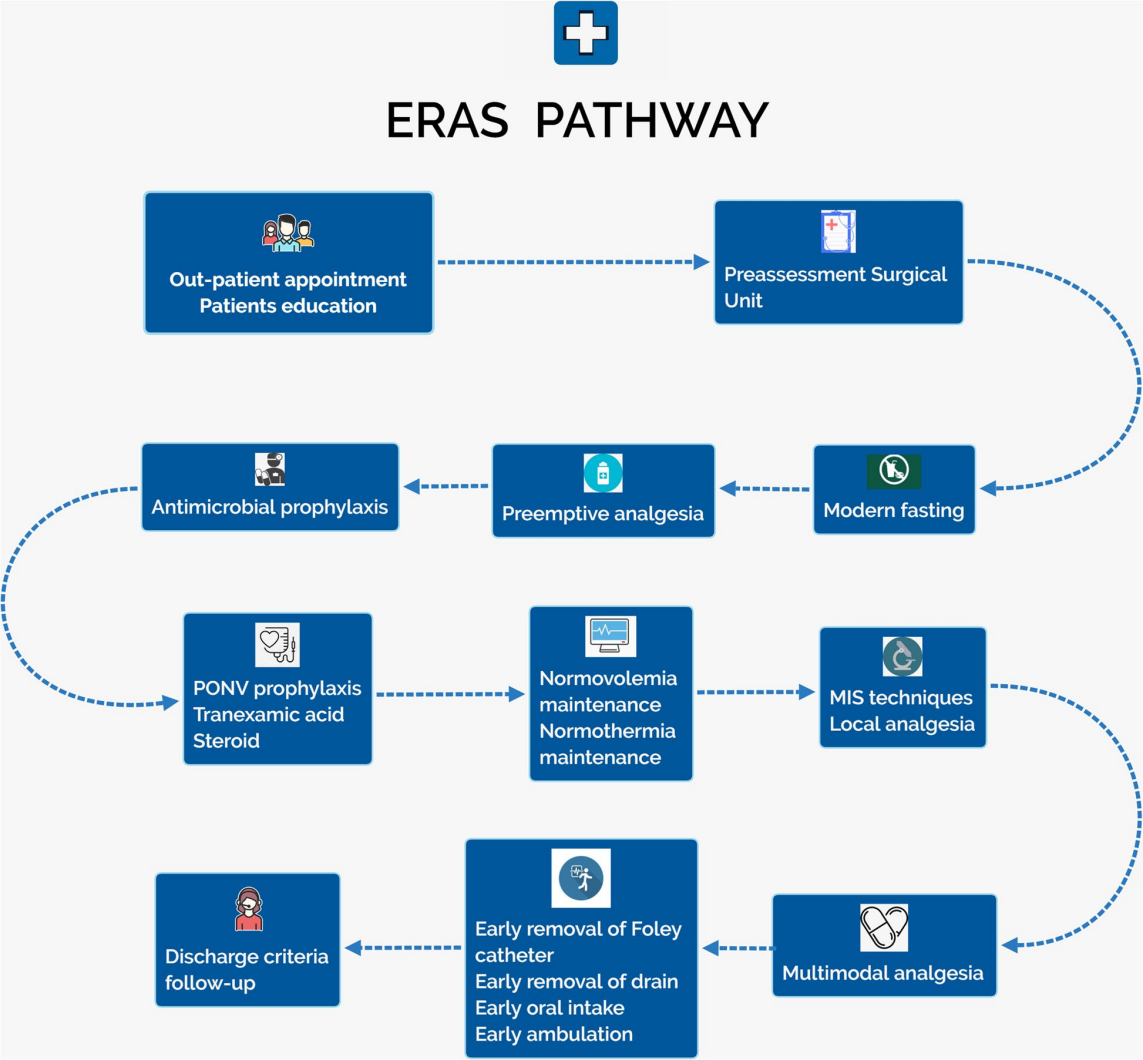
Overview of the patient path for ACDF according to ERAS procedure. Abbreviations: *ERAS* enhanced recovery after surgery, *PONV* postoperative nausea and vomiting, *MIS* minimally invasive surgery

The ERAS pathway was implemented through collaboration of experts from surgery, anesthesiology, nutrition, pharmacy, nursing, physical therapy and neurophysiology and hospital administrators. The patients have a central and proactive role during their ACDF treatment. The summary ERAS procedure was shown in the Fig. 1. Our ERAS pathway is made up of 4 phases: the preadmission, preoperative, intraoperative and postoperative phases (Table 1). The preadmission phase includes outpatient appointments, patient education and preassessment surgical unit (PASU) attendance. All patients are seen as outpatients in a formal clinic setting by the surgeon. The need for surgery, imaging and blood tests are carefully explained. Additional interventions include smoking cessation and medication modification and cessation. Patient education is led by surgery providers and fast-track nurses. A hand-out that details the ERAS aims, perioperative analgesia, modern fasting, surgical technique, rehabilitation goal discharge criteria and a follow-up plan is available for the patients in Chinese. An anesthetist at the PASU provides a consultation to ensure that the patient can undergo general anesthesia appropriately. The condition of the patient is reported to the surgeon by the anesthetist and nurse to optimize the treatment of the patient.

**Table 1:** The components of enhanced recovery after surgery and conventional recovery after surgery protocols

Phases	Components	ERAS Protocol	Conventional Protocol	Ref
**Preadmission**	Out-patient appointment	• Surgical decision validation • Imaging—X-rays, CT, MRI and neurophysiology if used • Medical history • Medication modification or cessation • Blood tests • Smoking cessation	Completed in hospitalization	[26–28]
	Patients education	• Surgical expectation setting provided by surgeon • ERAS education by fast-tracking nurses • Handout including ERAS aims, analgesia, modern fasting, surgical technique, Rehabilitation goals, discharge criteria and follow-up plan	General informed consent without ERAS education	[13]
	Preassessment Surgical Unit	• Anesthetist consultation • ASA • Feedback by nurse or anesthetist to surgeon	Not conventionally operated	[26]
**Preoperative**	Modern fasting	Solids until 6 h and clear liquids (CHO beverage, Outfast) until 2 h prior to surgery	Fasting 8 h	[29]
	Preemptive analgesia	Celecoxib (200 mg) and pregabalin (75 mg) given orally in the holding area	Not conventionally used	[30–33]
	Antimicrobial prophylaxis	Cefuroxime (1.5 g) given 30 min prior to incision	Not performed at a consistent time	[34]
**intraoperative**	PONV prophylaxis	5-HT receptor antagonist (ramosetron) administrated during anesthetic induction	No routinely administrated	[28]
	Tranexamic acid	1 g bolus prior to incision followed by 0.5 g/hour infusion	Not conventionally used	[35]
	Steroid	Intravenous dexamethasone (10 mg) given prior to incision	Not conventionally used	[27]
	Normovolemia maintenance	Intraoperative goal-directed fluid administration, administer vasopressors to support blood pressure control	Caregiver preference	[36]
	Normothermia maintenance	Core temperature was maintained above 36℃ by using convective warming device	Performed using blankets	[37]
	Foley catheter	Catheterization under anesthesia	Catheterization before anesthesia	[27]
	MIS techniques	• Microscope assisted surgery • Self-retaining retractors were used	No microscope Traction by assistants	[2, 30]
	Local analgesia	Local infiltration of incision at the end of surgery	Rarely used	[30]
**postoperative**	MMA	Opioid sparing, intravenous parecoxib 40 mg after surgery, celecoxib 200 mg and pregabalin 75 mg every 12 h as oral intake tolerated, intramuscular tramadol 100 mg if pain was poorly controlled	Caregiver preference	[30]
	Early ambulation	Handouts including mobilization methods and goals provided by caregivers, patients encouraged to get out of bed on POD 1	Not provided handouts, patients required to have bed rest on POD 1–3	[26, 27, 30]
	Early oral intake	Clear liquids permissible on POD 0. Patients encouraged to have oral diet at will after recovery from anesthesia	Not provided clear liquids	[30]
	Early removal of Foley catheter	Removing the Foley catheter at POD 1	Extraction time depends on clinicians	[27]
	Early removal of drain	POD 2	Clinicians' preference	[30]
	Discharge criteria	Mobilization with help; adequate pain control (NRS < 3), toleration of oral intake, normal body temperature, no wound infection; and no severe complications	Experience judgment of clinicians	[38]
	follow-up	• A mobile app was used for keeping contact with patients • Postoperative fixed time was followed up, including NRS, NDI and JOA scores	Patients went to the hospital for reexamination	[26]

*ERAS* enhanced recovery after surgery, *CHO* carbohydrate, *PONV* postoperative nausea and vomiting; 5-HT, 5-hydroxytryptamine, *MIS* minimally invasive surgery, *MMA* multimodal analgesia, *POD* postoperative day, *NRS* Numerical Rating Scale, *NDI* Neck Disabilitv Index, *JOA* Japanese Orthopaedic Association Scores

Preoperatively, solids within 6 h and carbohydrate beverages within 2 h prior to surgery are permissible for modern fasting. 200 mg of celecoxib, 75 mg of pregabalin and 1 g of acetaminophen are administered 1 h before surgery. Antimicrobial prophylaxis involves the use of 1.5 g of cefuroxime, given 30 min before incision. Intraoperative interventions include administration of tranexamic acid and steroids. A 5-HT receptor antagonist (ramosetron) is administered during anesthetic induction to prevent postoperative nausea and vomiting (PONV). A urinary catheter is placed under anesthesia. In addition, the surgery is assisted by an operating microscope and self-retaining retractors. After incision closure, the incision is infiltrated with a local anesthetic agent (5 mg/mL ropivacaine hydrochloride).

Evidence-based multimodal analgesia is carried out postoperatively. Early oral intake and ambulation are encouraged. Furthermore, the urinary catheter and drain are also promptly removed. Discharge criteria include mobilization without help, adequate pain control (NRS < 3), toleration of oral intake, normal body temperature, no wound infection, and no severe complications. Regarding the follow-up, a mobile app is used to keep contact with the patient after discharge.

### Surgical techniques

After anesthesia, the patient was placed in a supine position, prepared and draped. The Smith-Robinson approach via a transverse incision was used for access to the anterior cervical spine. In the ERAS group, all surgeons performed discectomy, foraminotomy and posterior decompression under a microscope. The posterior longitudinal ligament at the intervertebral disc (IVD) space was excised routinely. After endplate preparation, the PEEK cage was placed into the IVD space. Finally, anterior cervical plating was utilized.

### Outcome measures

The primary outcome was LOS. Secondary outcomes included cost, MacNab assessment for postoperative patient satisfaction, complication rate and 90-day readmission and reoperation. And perioperative factors and postoperative complications were reviewed.

### Statistical analysis

Continuous variables are summarized as the mean (standard deviation, SD) and median (range, R). Categorical variables are summarized as frequencies and percentages. To compare continuous variables between the ERAS and conventional groups, an independent samples t test or Wilcoxon rank-sum test was used. Categorical variables were compared between the ERAS group and the conventional group by using a chi-square test or Fisher’s exact test. All analyses were performed using SPSS software (version 19.0, IBM Corp., Armonk, NY, USA). *P* < 0.05 was considered statistically significant.

## Results

### Patient population

A total of 143 patients were included in this study, 73 of whom were in the conventional group and 70 of whom were in the ERAS group. In the conventional group, 37 patients underwent 1-level ACDF, and 28 patients underwent 2-level ACDF, 8 patients underwent 3-level ACDF. In the ERAS group, 52 patients underwent 1-level or 2-level ACDF, 12 patients underwent 3-level ACDF, and 6 patients underwent 4-level ACDF.

### Reduction of LOS and overall complication rate in ERAS Group

The demographic data and comorbidities of patients are shown in Table 2. There were no significant differences between the two groups in age, sex, comorbidities, body mass index (BMI) or American Society of Anesthesiologists (ASA) grade (*p* > 0.05, Table 2). More patients undergoing multi-level surgery (*n* ≥ 3) were included in the ERAS group (*p* < 0.05, Table 2). In addition, the lumbar vertebra bone mineral density (BMD) in the conventional group was significantly lower than that in the ERAS group (*p* < 0.05, Table 2). The LOS was significantly shorter in the ERAS group than in the conventional group. The median decreased from 5 to 4 days (*p* < 0.05, Table 3). The ERAS group had a shorter operative time and less surgical drainage at POD 1 than the conventional group, but these differences were not statistically significant (*p* > 0.05, Table 3). With respect to the total cost, an 9.5% reduction was shown in the ERAS group. This corresponds to an average of ¥6151.24 in savings, but this was no statistically significant. What's more, the MacNab assessment for postoperative patient satisfaction in the ERAS group was better than that in conventional group (*p* < 0.05, Table 3).

**Table 2:** Demographic and baseline characteristics of ACDF patients

Parameter	Convention (*n* = 73)	ERAS (*n* = 70)	*p* value
Age(years), mean ± SD	52.07 ± 10.6	53.2 ± 9.3	0.499
Gender (male/female)	36/37	39/31	0.444
BMI (kg/m^2^), mean ± SD	24.65 ± 3.2	23.94 ± 3.0	0.165
ASA grade (n)			0.442
-ASA 1	0	2	
-ASA 2	57	58	
-ASA 3	7	10	
Levels (n)			0.019
-1	37	24	
-2	28	28	
-3	8	12	
-4	0	6	
Lumbar vertebra BMD (T), mean ± SD	-2.42 ± 1.1	-1.76 ± 1.2	0.013
Diabetes mellitus (n)	1	4	0.338
Hypertension (n)	11	12	0.736
Chronic cardiovascular disease (n)	2	1	1.00

*ACDF* anterior cervical discectomy and fusion, *BMI* body mass index, *ASA* American Society of Anesthesiologists, *BMD* bone mineral density

**Table 3:** Perioperative factors and postoperative outcomes

Parameter	Convention (*n* = 73)	ERAS (*n* = 70)	*p* value
LOS, median (range)	5(3–8)	4(3–11)	0.002
Cost, median (range)	64,941.72(48,066.31–94,072.60)	58,790.48(41,350.02–114,729.62)	0.376
Operative time (minutes), median (range)	135(55–400)	132.5(44–315)	0.739
Drainage at POD 1, median (range)	30(1–140)	20(2–120)	0.232
MacNab (n)			0.001
Excellent	41	59	
Good	29	10	
Fair	3	1	
Poor	0	0	
Major complications	13(17.8%)	3(4.3%)	0.010
Prolonged dysphagia	3(4.1%)	2(2.9%)	1.000
Hardware failure, n(rate)	9(12.3%)	1(1.4%)	0.026
Dyspnea, n(rate)	1(1.4%)	0	
Minor complications	19(26.0%)	7(10.0%)	0.013
Dysphagia/dysphonia, n(rate)	15(20.5%)	6(8.6%)	0.043
Nausea and vomiting, n(rate)	4(5.5%)	1(1.4%)	0.367
Overall complications, n(rate)	32(43.8%)	10(14.3%)	0.000
90-day readmission (n)	0	0	
90-day reoperation (n)	0	0	

*LOS* length of Stay. *POD* postoperative day

All surgical complications were reviewed in detail (Table 3). The rate of overall complications was significantly higher in the conventional group than in the ERAS group. We have classified surgical complications into major and minor complications. The major complications included prolonged dysphagia, hardware failure and dyspnea. The minor complications included dysphagia/dysphonia, nausea and vomiting. The rates of dysphagia/dysphonia in minor complications and hardware failure in major complications were lower in the ERAS group than in the conventional group (*p* < 0.05, Table 3).The rate of nausea and vomiting was also reduced in ERAS group (*p *> 0.05, Table 3). There was no significant difference in prolonged dysphagia between two groups (*p* > 0.05, Table 3). One case in the conventional group involved dyspnea after surgery. Furthermore, there were no 90-day readmission and reoperation in either group.

## Discussion

ACDF is the most common surgery for the treatment of degenerative cervical disorders. As the demand for anterior cervical fusion grows rapidly, more older patients with comorbidities have undergone ACDF than in previous years. Meanwhile, LOS, cost, rates of complications and risk of readmission have increased significantly [18–20]. ERAS aims to reduce the stress response to surgery by implementing evidence-based interventions. Therefore, this emerging technique is also applicable to ACDF surgery. Herein, we reported that implementation of an ERAS pathway for ACDF in our hospital has significantly decreased the LOS, cost, and improved patient satisfaction without increasing 90-day readmission and reoperation rates. Furthermore, ERAS decreased in postoperative complications.

To date, few studies have reported the establishment and implementation of an ERAS for ACDF surgery as well as relevant outcomes. Debono et al. compared outcomes after ACDF before and after ERAS pathway implementation. Introduction of the ERAS approach was associated with a decreased LOS and increased patient satisfaction. There were no significant differences in overall complications, 90-day reoperation or 90-day readmission [26, 39]. In a retrospective cohort study, 33 patients were cared for under an ERAS pathway tailored for ACDF and followed up to postoperative day (POD) 90. The results showed that the pathway was associated with a shorter LOS, minimal complications, and no readmissions within 90 days of surgery [40]. In 2020, a study reviewed their ACDF-ERAS pathway case series retrospectively. The outcomes of these cases support the safety of the application of the ERAS pathway to ACDF patients [27]. However, ERAS is still emerging for anterior cervical fusion. Further studies are needed to confirm the potential positive influence of ERAS on anterior cervical spine surgery.

We previously reported our experience with an ERAS pathway for minimally invasive transforaminal lumbar interbody fusion [13]. Based on the templated ERAS pathway, we reviewed the evidence for interventions that have a positive influence on outcomes after ACDF surgery and established a tailored pathway. Local medical resources and culture were also taken into consideration. Our ACDF-ERAS pathway consisted of 4 chronologic phases: the preadmission, preoperative, intraoperative and postoperative phases. A study has reported that patients who receive sufficient counseling have higher levels of satisfaction than those who receive insufficient education [14, 41]. To ensure a proactive role for patients in their perioperative management, patient education and anesthesia consultation were advocated. Furthermore, several well-established ERAS components, including carbohydrate treatment, preemptive analgesia and antimicrobial prophylaxis, were implemented preoperatively [28, 41].

In this study, the rate of overall complications was significantly lower in the ERAS group than that in the conventional group. This may be related to our components in ERAS protocol. PONV is a common problem in patients following general anesthesia. Herein, a 5-HT receptor antagonist and dexamethasone were administered intraoperatively as a regular antimetic regimen. Moreover, dexamethasone has been found to reduce postoperative dysphagia and prevertebral soft swelling in anterior cervical spine surgery [42]. Although the results showed a significant difference of hardware failure rate between the two groups, It might be attributed to the difference of BMD between the two groups rather than EARS protocol. Intraoperative use of microscopy is strongly recommended. Microscopy-assisted ACDF has been shown to reduce blood loss, postoperative pain and complications [43, 44]. Catheterization was performed under general anesthesia to avoid embarrassment and to reduce discomfort. At the end of surgery, local infiltration of the incision was utilized to produce postsurgical analgesia [45]. Several surgical routines, such as postoperative analgesics, the timing of ambulation and oral intake, the duration of drainage and catheterization and the timing of discharge, vary greatly and significantly affect outcomes. In our pathways, which were mentioned above were standardized and protocolized. As a result, the fluidity of the patient pathway was optimized. It is worth noting that after discharge, the use of mobile app allowed for online personalized monitoring and follow-up. Through the app, patients were able to receive rehabilitation guidance from doctors at any time, and the rehabilitation of patients was assessed every six months. The components mentioned above contributed to improving patient satisfaction.

There are numerous factors that affect LOS, including postoperative complications, preoperative comorbidities, and the timing of ambulation. Decreased LOS is a crucial outcome measure in many ERAS pathways. In our ERAS protocol, the urinary catheter and wound drainage were removed in the morning of POD 1 and POD 2, which helped patients get out of bed on POD 1. In the conventional care group, the removal of each was determined by the surgeon's experience and preference. Additionally, the discharge criteria were clearly defined. This contributes to reducing LOS because traditionally in China, longer hospitalization means better recovery from injury to the musculoskeletal system. However, the mean difference of LOS was relatively small due to two reasons. Firstly, to this day, it is difficult for patients to quickly obtain emergency medical resource in the community of China. Postoperative complications could lead to litigation. As a result, hospitalization for one or even several days is still the rule in China. Especially, to minimize the risk of fatal complications, a postoperative monitoring period in the surgical intensive unit at least one night is indispensable for the ACDF patients. The safety of patients before discharged must be ensured. Therefore, ambulatory procedure or out-patient procedure are not applicable to the ACDF patients in China. On the other hand, the patients undergoing multi-level ACDF surgery in ERAS group was significantly higher than those in conventional group. Further multidisciplinary work on the implementation of more optimized ERAS pathway should be performed by us. Notably, decreased postoperative complications contributed to a significantly lower LOS. The incidence of postoperative dysphagia/dysphonia decreased significantly after ERAS implementation. This probably resulted from the use of microscopy and steroids (Table 1, intraoperative).

The median cost was reduced from 64,941.72 to 58,790.48 (*p* > 0.05, Table 3). However, the difference was not statistically significant. A major reason is that the patients undergoing multi-level ACDF surgery in ERAS group was significantly higher than those in conventional group ((*p* < 0.05, Table 2). As widely known, the multi-level ACDF surgery needs more implants and longer surgery time [27]. Therefore, to eliminate the factor, we will design a prospective study on ACDF.

The study has significant limitations. Due to the historical adoption of ERAS in our institution, our study was retrospective. This design was a compromise. The two cohorts were from different time frames, which resulted in recall bias and selection bias. In this regard, we have reviewed the data objectively to ensure the accuracy of the results. The number of multi-level surgery patients in ERAS group was significantly higher than that in conventional group. Moreover, the sample size of the study was small, which may be one of the reasons why the cost reduction is not statistically significant. As a result, the strength of evidence with respect to the effectiveness and safety of our ERAS pathway is restricted. This is also a common limitation of retrospective studies. Therefore, clinical randomized controlled trials based on this study will be designed to find more powerful evidence. Second, long-term follow-up data, including the numeric rating scale for pain intensity, Oswestry Disability Index, and patient satisfaction, were not obtained. The long-term effects of ERAS remain unknown. Finally, the universal applicability of this ERAS pathway should be considered cautiously because of the variations in resources, volume of surgical procedures, and surgical training levels among different surgeons, settings, locales, and hospitals.

## Conclusion

This study describes our ERAS protocol for ACDF, focusing on facilitating safe and enhanced recovery. The ERAS pathway for ACDF provides reductions in LOS, cost and complication rates, and an increase in patient satisfaction. Meanwhile, the ERAS pathway did not affect 90-day readmission and reoperation. In summary, the ERAS pathway promotes rapid recovery of patients from ACDF effectively and safely.

## Data Availability

All data are fully available without restriction. The database used in this study is available from the corresponding author on reasonable request.

## Abbreviations

ACDF: Anterior cervical discectomy and fusion
LOS: Length of stay
PASU: Preassessment surgical unit
PONV: Postoperative nausea and vomiting
NRS: Numerical rating scale
IVD: Intervertebral disc
CHO: Carbohydrate
5-HT: 5-Hydroxytryptamine
MIS: Minimally invasive surgery
MMA: Multimodal analgesia
POD: Postoperative day
NDI: Neck Disabilitv Index
JOA: Japanese Orthopaedic Association Scores
BMI: Body mass index
ASA: American Society of Anesthesiologists
SICU: Surgical intensive unit
BMD: Bone mineral density
